# Association between skipping breakfast and prediabetes among adolescence in Japan: Results from A-CHILD study

**DOI:** 10.3389/fendo.2023.1051592

**Published:** 2023-02-22

**Authors:** Keitaro Miyamura, Nobutoshi Nawa, Aya Isumi, Satomi Doi, Manami Ochi, Takeo Fujiwara

**Affiliations:** ^1^Department of Global Health Promotion, Tokyo Medical and Dental University, Tokyo, Japan; ^2^National Institute of Public Health, Department of Health and Welfare Services, Saitama, Japan

**Keywords:** skipping breakfast, type 2 diabetes, prediabetes, adolescent, HbA1c

## Abstract

**Objective:**

Adolescents with prediabetes are at high risk of developing type 2 diabetes in later life. It is necessary to identify risk factors for prediabetes in adolescents. This study aimed to examine the association between skipping breakfast and prediabetes among adolescents in Japan.

**Study design:**

We used the population-based cross-sectional data of eighth grade in junior high school students from the Adachi Child Health Impact of Living Difficulty (A-CHILD) study conducted in Adachi City, Tokyo, Japan, in 2016, 2018, and 2020. Skipping breakfast was assessed using self-reported questionnaires (N=1510). Prediabetes was defined as hemoglobin A1c (HbA1c) levels of 5.6-6.4%. The association between skipping breakfast and prediabetes was evaluated using multivariate logistic regression analysis. Stratified analysis was also performed using BMI, 1 SD or more, or less than 1SD, as overweight was defined as 1SD or more.

**Results:**

Students who skipped breakfast were 16.4% (n=248). The prevalence of prediabetes was 3.8% (n=58). Skipping breakfast exhibited a significant association with prediabetes (OR:1.95, 95% CI: 1.03 to 3.69) after adjusting for sex, annual household income, family history of diabetes mellitus, BMI, and survey year. Stratified analysis showed stronger association among students with overweight (BMI ≥1SD) (OR=4.31, 95% CI 1.06-17.58), while non-sigificant among students without overweight (BMI<1SD) (OR=1.62, 95% CI 0.76-3.47).

**Conclusions:**

Skipping breakfast in Japanese adolescents, especially those with overweight, was associated with prediabetes. The promotion of avoiding skipping breakfast may help to prevent prediabetes.

## Introduction

Type 2 diabetes is an emerging and unsolved global health problem. Recent studies reported that the prevalence of type 2 diabetes in adults worldwide was about 8%, and the incidence of diabetes are plateauing (1–3). Type 2 diabetes can lead to blindness, dialysis, and cardiovascular disease, significantly impairing patients’ quality of life (1). The prevalence of patients with young-onset type 2 diabetes is increasing worldwide (4, 5), and mortality and cardiovascular morbidity associated with type 2 diabetes differed significantly by age at diagnosis, with mortality and cardiovascular morbidity being highest among patients with early-diagnosed type 2 diabetes (6). To prevent type 2 diabetes, there is a need to identify risk factors in early-stage, including adolescents with prediabetes (7).

To identify possible risk factors for prediabetes in adolescence, the risk factors for type 2 diabetes would be the most prominent. For example, sedentary lifestyle or lack of physical exercise (8), improper dietary intakes (9), obesity, and family history of diabetes are well documented as risk factors for type 2 diabetes (8). Among them, we focus on skipping breakfast as a risk factor for prediabetes in adolescence because it is prevalent among adolescents, for example, 8.0% in junior high school in Japan (10, 11). Previous studies have suggested robust biological mechanisms in the association between skipping breakfast and prediabetes. Skipping breakfast could affect glucose metabolism by elevating free fatty acid level (12) and disrupting circadian rhythms (13). Furthermore, skipping breakfast can be associated with increased appetite (12) and poor diet (14). In addition, skipping breakfast may also decrease physical activity in the morning (15, 16).

A few cross-sectional studies showed that skipping breakfast was associated with elevated fasting glucose levels in childhood (aged 6-17 years old) (17, 18). However, population-based studies of adolescents in Asian populations are lacking. Considering the biological mechanisms of the effects of skipping breakfast on glucose metabolism, racial differences in insulin sensitivity and insulin response (19) may result in racial differences in the risk of skipping breakfast. In a European population-based study, significant differences among breakfast consumption habits and fasting blood glucose were seen only in boys (18). In a Brazilian study, a higher frequency of eating breakfast was negatively correlated with fasting blood glucose levels (17). However, it may be difficult to generalize the results because researchers investigated only children with obesity, with the subjects recruited *via* television commercials and newspaper advertisements. Conversely, a study among primary school children in Taiwan reported no association between skipping breakfast and prediabetes using fasting glucose levels (20). However, it may be too early to assess the associations because insulin resistance increases during adolescence (21).

The effect of skipping breakfast on glucose metabolism may be even higher in children with obesity because obesity increases insulin resistance and the risk of glucose intolerance (22). In individuals without obesity, skipping breakfast may decrease total daily energy intake. In contrast, in individuals with obesity, skipping breakfast may increase energy intake in the second half of the day without decreasing total energy intake (15, 16, 23). In other words, obesity may be an effect modifier in the association between skipping breakfast and diabetes risk. In addition, it has been reported that Asians are more likely to accumulate visceral fat even at the same BMI and to develop diabetes even with mild obesity compared to Whites (24). Therefore, it is also essential to evaluate the possibility that children with overweight may be a high-risk group.

In this research, we used a set of population-based data of junior high school children (aged 13–14 years old) from the Adachi Child Health Impact of Living Difficulty (A-CHILD) study in Tokyo, Japan, collected in 2016, 2018, and 2020. This study aimed to examine the association between skipping breakfast and prediabetes during adolescence in Japan and whether overweight status modify the association.

## Methods

### Study design and subjects

We used the cross-sectional data from the A-CHILD study conducted in Adachi City, Tokyo, Japan, in 2016, 2018, and 2020 (25–27). Details of this study protocol can be found somewhere (27). This study was approved by the Ethics Committee at the National Center for Child Health and Development (Study ID: 1147) and Tokyo Medical and Dental University (Study ID: M2016-284). Self-reported questionnaires with unique anonymous ID were administered to children in representative junior high schools (13-14 years old) in October 2016, 2018, and 2020. Children and their parents answered questionnaires at home and returned the questionnaires to their schools. Children responded to questions about lifestyle, while parents responded to questions about the family environment and their medical history. In 2016, 588 questionnaires were collected (77.9% return rate), in 2018, 583 questionnaires were collected (86.2% return rate), and in 2020, 551 questionnaires were collected (83.6% return rate), for a total of 1722 questionnaires collected (82.4% return rate). Questionnaire responses were linked to school health checkup data for body mass index (BMI) and blood test data conducted in Adachi City including HbA1c levels. Student participation in the health checkups was voluntary. The overall participation rate for health checkups was 75.4%, with 66.5% in 2016, 82.0% in 2018, and 79.0% in 2020.

**Figure 1:**
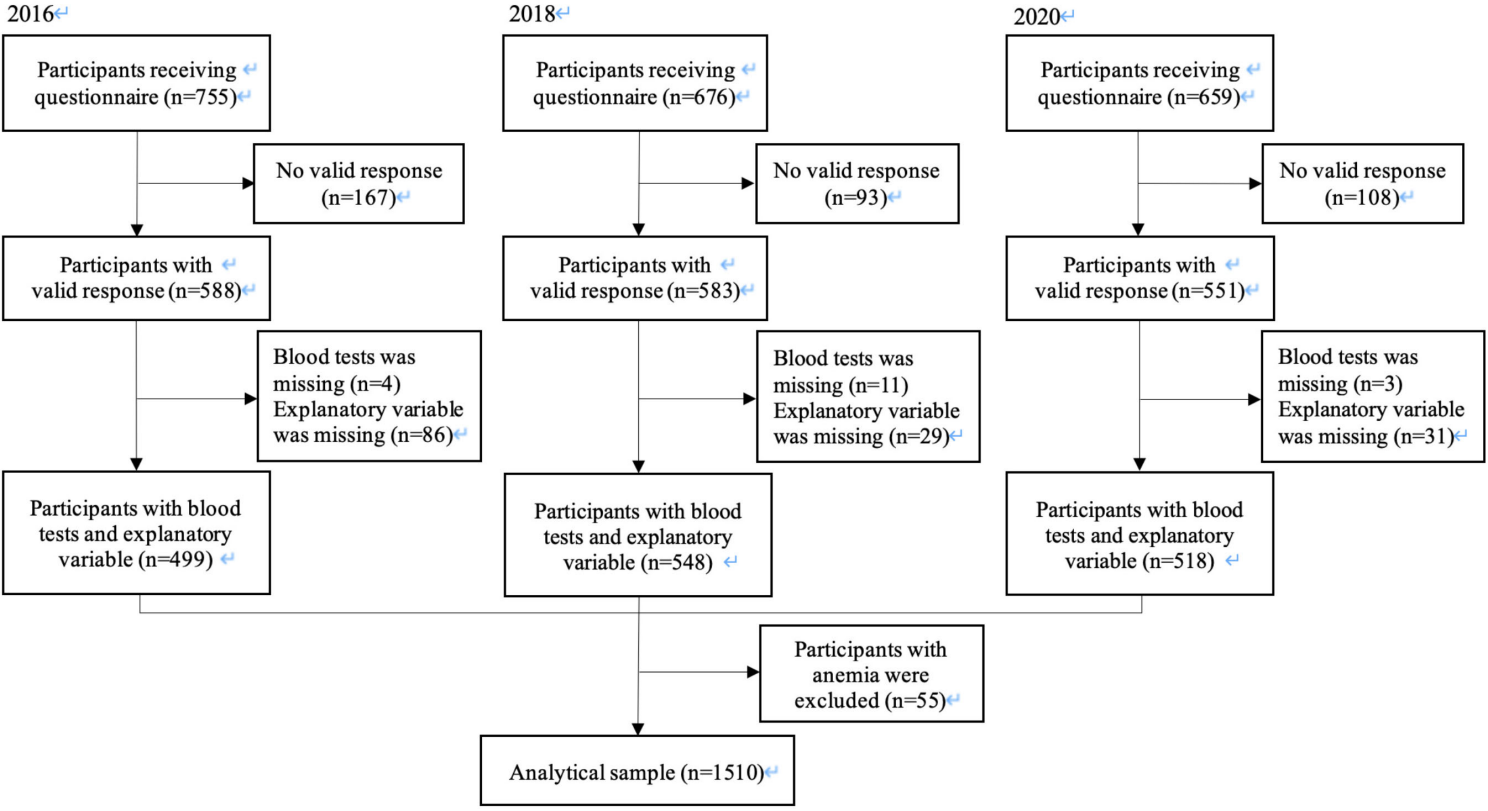
Participant flow chart. Of the 2090 2nd year students in seven representative junior high schools in Adachi City in 2016, 2018 and 2020, we analyzed 1510 students, who provided data for the frequency of breakfast and the blood tests.

Parents or children who did not respond, who left all answers blank, who did not agree to participate in the study, or whose children did not receive school checkups were excluded as invalid responses, and the remaining respondents were considered valid. Children who had missing data about the frequency of breakfast or HbA1c value were excluded. Children with anemia (defined as less than 12.0 g/dl of hemoglobin levels (28)) were also excluded because chronic anemia such as iron deficiency anemia elevates HbA1c level due to the effect of erythrocyte turnover although blood glucose does not elevate (29). The analysis was carried out using the data of 1510 participants (**Figure 1**).

### Skipping breakfast

Skipping breakfast was assessed using the following question “How often do you eat breakfast per week?” based on previous studies (18, 20). The responses to this question were “every day,” “sometimes,” “rarely,” or “never.” To compare those who eat breakfast every day with those who do not eat breakfast every day, we collapsed four categories into two: “every day” or “sometimes/rarely/never”.

### Prediabetes

In this study, we evaluated HbA1c levels of 5.6-6.4% as prediabetes because the Japanese Diabetes Diagnostic Criteria Review Committee considers HbA1c levels of 5.6-6.4% as a group at high risk of developing diabetes in the future (30). We took a venipuncture blood sample from the arm at the laboratory and measured HbA1c level using an enzymatic assay. The students were not required to fast prior to having the blood test.

### Covariates

Breakfast habits and risk of prediabetes are affected by demographic factors and socioeconomic status (8, 31). We chose child sex, annual household income as socioeconomic status, family history of diabetes, BMI, and survey year, as covariates, based on previous studies (17, 18, 20). Annual household income was categorized into four groups (<3.0 million yen, 3-6 million yen, 6-10 million yen, ≥10 million yen) based on the previous study (25). Family history of diabetes was categorized as “yes” when mother or father of participants had diabetes, and “no” when both mother and father of participants did not have diabetes. Children’s BMI was calculated from their height and weight and assessed by z-scores based on the WHO Child Growth Standards according to age and sex, which can be applied to Japanese (32). BMI was categorized into three groups (<-1SD, -1SD to1SD, ≥1SD).

Items related to lifestyle habits other than breakfast habits were also investigated, such as sleep and exercise habits. Wake-up time was categorized into three groups (< 1 time/week, 1-2 times/week, ≥3 times/week). Sleep duration was calculated from the difference between waking and sleeping times for each hourly category because we did not ask about sleep duration. For example, the “7:00 - 8:00 a.m.” wake-up time category was considered as 7:30, and the “after 24:00” bedtime category was considered as 24:30. If the person went to bed at 1:00, his/her sleep duration could have been overestimated. Sleep duration was categorized into four groups (≤ 6hours, 7hours, 8-10 hours, ≥11 hours) based on a consensus statement of the American Academy of Sleep Medicine (33). The frequency of exercise was categorized into three groups (< 1 time/week, 1-2 times/week, ≥3 times/week). Missing data with all covariates, which was adjusted for regression analysis, was created as a new dummy variable.

### Statistical analysis

The association between skipping breakfast and prediabetes was evaluated using logistic regression analysis to calculate crude and adjusted odds ratio (OR) with 95% confidence intervals (CI). Sex, socioeconomic status, family history of diabetes, BMI, and survey year were put in the adjusted model. The VIF for the wake-up time variable was about 1 to 2, suggesting that there was no multicollinearity (34) (**Supplementary Table 1**). Thus, we performed the logistic regression analyses further adjusted for wake-up time and frequency of exercise. Furthermore, previous studies have shown that it is not the wake-up time but sleep duration (35, 36) and sleep disturbances (35) that affects diabetes. We also performed an analysis adjusted for sleeping time instead of wake-up time.

We also evaluated the effect of the interaction of overweight/obesity (BMI ≥ 1 SD (37)) on the association of skipping breakfast with prediabetes. Considering that estimating interactions requires a larger sample size than estimating main effects (38), the interaction term indicated a weak but possible effect modification (p-value for interaction 0.21), even though the interaction p-value is slightly larger (39). In other words, the effect of frequency of breakfast on prediabetes could vary depending on the presence or absence of overweight, we conducted stratified analysis by BMI ≥1 SD (i.e., students with overweight) and BMI <1 SD (i.e., students without overweight). Data analyses were carried out using STATA version 15 (Stata Corp LP, College Station, TX, USA).

## Results

The proportion of students who ate breakfast every day and sometimes/rarely/never were 83.6% and 16.4%, respectively. The prevalence of prediabetes was 3.8%. There were no students whose HbA1c level was more than 6.5%, which is one of the diagnostic criteria of diabetes (American Diabetes Association 2010). There was no large change in the percentage of students who ate breakfast daily and in the prevalence of prediabetes between survey years. (**Supplementary Table 2**). The proportion of boys and girls were similar. A total of 12.3% had an annual household income of fewer than 3million yen. The percentage of girls who did not have breakfast every day (19%) was greater than that of boys who did not have breakfast every day (14%) (**Table 1**).

**Table 1:** Characteristics of participants (N=1510).

		Total	Frequency of breakfast	
			Every day	Sometimes/rarely/never
		(N=1510)	(N=1262; 83.6%)	(N=248; 16.4%)
		N (%)	N (%)	N (%)
Prediabetes	HbA1c<5.6	1452 (96.2%)	1218 (96.5%)	234 (94.4%)
	HbA1c≧5.6	58 (3.8%)	44 (3.5%)	14 (5.6%)
Child Sex	Boy	757 (50.1%)	651 (51.6%)	106 (42.7%)
	Girl	753 (49.9%)	611 (48.4%)	142 (57.3%)
	Missing	0 (0%)	0 (0%)	0 (0%)
Annual household income (million yen)	< 3	185 (12.3%)	141 (11.2%)	44 (17.7%)
	3 - 6	471 (31.2%)	400 (31.7%)	71 (28.6%)
	6 - 10	500 (33.1%)	434 (34.4%)	66 (26.6%)
	≥ 10	137 (9.1%)	122 (9.7%)	15 (6.0%)
	Unknown/Missing	217 (14.4%)	165 (13.1%)	52 (21.0%)
Family history of diabetes	No	1440 (95.4%)	1204 (95.4%)	236 (95.2%)
	Yes	70 (4.6%)	58 (4.6%)	12 (4.8%)
	Missing	0 (0%)	0 (0%)	0 (0%)
BMI	<-1SD	262 (17.4%)	227 (18.0%)	35 (14.1%)
	-1SD to 1SD	991 (65.6%)	833 (66.0%)	158 (63.7%)
	≥1SD	228 (15.1%)	178 (14.1%)	50 (20.2%)
	Missing	29 (1.9%)	24 (1.9%)	5 (2.0%)
Survey year	2016	483 (32.0%)	405 (32.1%)	78 (31.5%)
	2018	525 (34.8%)	435 (34.5%)	90 (36.3%)
	2020	502 (33.2%)	422 (33.4%)	80 (32.3%)

**Table 2** shows the odds ratio (OR) of skipping breakfast for prediabetes. Students who did not eat breakfast every day were 1.66 times more likely to have prediabetes than those who ate breakfast every day in the crude model (OR: 1.66, 95% CI: 0.89 to 3.07). After adjusting for child sex, annual household income, family history of diabetes, skipping breakfast showed significant association with prediabetes (OR:1.95, 95% CI: 1.03 to 3.69) (Adjusted model). The OR of skipping breakfast to annual household income determined using univariate logistic regression was a negative association (OR: 0.39, 95% CI: 0.21 to 0.74 (“≥ 10 million yen” with reference to “<3million yen”)), whereas that of prediabetes to annual household income determined using univariate logistic regression was a positive association (OR: 1.36, 95% CI: 0.33 to 5.5 (“≥ 10 million yen” with reference to “<3million yen”)). Thus, annual household income was a negative confounder (40), leading to an underestimation of its effect. For this reason, the OR increased after adjusting for annual household income in the adjusted model.

**Table 2:** Odds ratio for prediabetes and skipping breakfast (n=1510).

		Crude		Adjusted model	
		OR	95%CI	OR	95%CI
Frequency of breakfast	Everyday	Ref		Ref	
	Sometimes/rarely/never	1.66	(0.89, 3.07)	**1.95**	**(1.03, 3.69)**
Child sex	Boy	Ref		Ref	
	Girl	0.56	(0.32, 0.96)	0.54	(0.31, 0.94)
Annual household income (million yen)	< 3	Ref		Ref	
	3 - 6	1.39	(0.45, 4.27)	1.45	(0.47, 4.48)
	6 - 10	2.99	(1.04, 8.59)	3.10	(1.07, 8.98)
	≥ 10	1.36	(0.33, 5.54)	1.42	(0.35, 5.83)
	Unknown/Missing	1.07	(0.28, 4.03)	1.09	(0.28, 4.14)
Family history of diabetes	No	Ref		Ref	
	Yes	1.56	(0.55, 4.42)	1.25	(0.43, 3.67)
BMI	<-1SD	0.87	(0.42, 1.82)	0.84	(0.40, 1.76)
	-1SD to 1SD	Ref		Ref	
	≥1SD	1.12	(0.55, 2.28)	1.00	(0.48, 2.09)
Year	2016	Ref		Ref	
	2018	1.02	(0.54, 1.92)	1.02	(0.54, 1.95)
	2020	0.91	(0.47, 1.75)	0.84	(0.43, 1.64)

OR, Odds ratio; CI, confidence interval.

Adjusted model: Adjusted for child sex, annual household income, family history of diabetes, BMI, and survey year.

Values in bold indicate statistically significant results.

**Table 3** shows the odds ratio (OR) of skipping breakfast for prediabetes stratified by BMI. Among students with overweight (BMI≥1SD), skipping breakfast showed stronger association with prediabetes in the adjusted model (OR: 4.31, 95% CI: 1.06, 17.58). In contrast, among students without overweight (BMI<1SD), skipping breakfast was not statistically significantly associated with prediabetes in the adjusted model (OR: 1.62, 95% CI: 0.76, 3.47).

**Table 3:** Odds ratio for prediabetes and skipping breakfast stratified by BMI .

	Frequency of breakfast	Crude		Adjusted model	
		OR	95%CI	OR	95%CI
BMI <1SD	Everyday	Ref		Ref	
	Sometimes/rarely/never	1.28	(0.61, 2.69)	1.62	(0.76, 3.47)
BMI ≥1SD	Everyday	Ref		Ref	
	Sometimes/rarely/never	**3.84**	**(1.07, 13.86)**	**4.31**	**(1.06, 17.58)**

OR, Odds ratio; CI, confidence interval.

Adjusted model: Adjusted for child sex, annual household income, family history of diabetes, BMI, and survey year.

Values in bold indicate statistically significant results.

The proportion of those who skipped breakfast was higher among those who woke up late, slept longer on weekdays, and infrequently exercised (**Supplementary Table 3**). In univariate analysis, breakfast skipping was significantly more frequent when waking up late, sleeping longer, and exercising less frequently (**Supplementary Table 4**). The logistic regression analysis with additional adjustments for wake-up time and exercise frequency, skipping breakfast remained significantly associated with prediabetes (OR: 2.01. 95%CI: 1.04, 3.89) (**Supplementary Table 1**). The logistic regression analysis adjusted for sleeping time instead of wake-up time showed similar results (OR: 1.98. 95%CI: 1.04, 3.79) (**Supplementary Table 5**).

## Discussion

This study investigated the association between breakfast habits and prediabetes using HbA1c levels in Japanese adolescents. We found that skipping breakfast was associated with prediabetes in adolescents, and this association was stronger among students with overweight.

To our knowledge, this is the first study to investigate the association between skipping breakfast and prediabetes in adolescents in the Asian population. A few cross-sectional studies showed the association between skipping breakfast and fasting glucose levels on a continuous scale in childhood (17, 18). However, these studies did not examine the association with prediabetes using specified cutoff for blood glucose or HbA1c levels. Our results suggest that skipping breakfast is also associated with prediabetes as measured by HbA1c levels, in addition to being associated with elevated blood glucose levels. In addition, our results suggest that the effect of skipping breakfast on glucose metabolism was greater among students with overweight.

The various biological mechanisms explaining the association between skipping breakfast and prediabetes can be speculated. As fasting conditions prolonged, energy sources are supplied by not only gluconeogenesis and degradation of glycogen but also lipolysis, leading to elevated levels of free fatty acid (FFA) (41). For example, FFA levels before lunch in those who skip breakfast is higher than in those who consume breakfast (12). Since the elevated FFA levels affect glucose metabolism by disrupting insulin receptor signaling in skeletal muscle and liver, elevated FFA levels by skipping breakfast may play an important role in developing insulin resistance (42).

Another potential biological mechanism is the disruption of the circadian clock, which normally controls the activity of enzymes and hormones associated with glucose metabolism. The central circadian clock, which is located in the suprachiasmatic nucleus of the hypothalamus, mainly responds to the external light-dark cycle (43), and the peripheral clocks located in peripheral tissues such as β-cells, muscles, adipose tissues, and the liver mainly respond to meal timing and content (15, 44). Asynchrony of the central and the peripheral circadian clocks was associated with reducing insulin and glucagon-like peptide 1 (GLP-1) secretion (45), insulin resistance, β-cell proliferation, and β-cell apoptosis (44). Randomized controlled trials reported that skipping breakfast affects clock and clock-controlled gene expression (13), and those who skip breakfast exhibit greater glucose of area under the curve and glucose variability after lunch than healthy, lean adults who consume breakfast (13, 15). In addition, lower transcript levels of clock genes such as Bmal1, PER1, and PER3 were inversely correlated with HbA1c levels (46). In other words, the disruption of the circadian clock due to skipping breakfast may affect insulin secretion and other factors, causing an increase in postprandial blood glucose, leading to an increase in HbA1c, i.e., the risk of prediabetes.

Obesity persistently increases plasma FFA levels both in the basal state and after glucose loading, and is a major contributor to insulin resistance (47). Insulin resistance cause hyperinsulinemia to maintain normoglycemia. Hyperinsulinemia can maintain normal blood glucose levels to some degree; however, chronic progressive insulin resistance and compensatory insulin hypersecretion can be beta cell stress and eventually to beta-cell failure, leading to prediabetes and then to type 2 diabetes (48). When individuals with overweight skip breakfast, insulin resistance can be further increased. Blood glucose levels after lunch in individuals with overweight may be even higher than in individuals without overweight due to inadequate compensatory insulin secretion for the elevation of insulin resistance. Since the lower the HbA1c level, the higher the contribution of postprandial blood glucose to the HbA1c level than fasting blood glucose (49), prediabetes assessed by HbA1c levels may capture the effect of skipping breakfast on the postprandial glucose level.

Based on current findings, skipping breakfast can be a risk factor for impaired glucose metabolism, leading to prediabetes. Therefore, breakfast consumption might be effective in modulating insulin sensitivity and secretion and reducing the risk of prediabetes. Breakfast consumption may be recommended, especially for people with obesity. Breakfast intake could not affect weight gain (23). However, it is necessary to pay attention to eating habits other than breakfast so that eating breakfast does not lead to excessive daily caloric intake. Moreover, it is important to intervene targeting to parents at an earlier age to establish the habit of consuming breakfast daily because dietary patterns could be established between 1 and 2 years old and continue into young adulthood (50).

Several limitations of this study should be acknowledged. First, a self-reported questionnaire on skipping breakfast could result in recall bias. Moreover, individuals may have had different understandings of the options for breakfast frequency, as we did not provide specific explanations. However, since the breakfast categories were divided into “every day” and “sometimes/rarely/never,” there would be unlikely misclassifications. Second, breakfast was not defined by period since wake-up or a time frame in the morning. However, breakfast time on weekdays among junior high school students would not be very different. Third, we were unable to include blood glucose levels to diagnose prediabetes. Fourth, we were unable to assess the pubertal stage like the tanner stage of each student, although the effects of glucose metabolism may vary by tanner stage (51). Fifth, we were not able to exclude other specific types of diabetes, such as type 1 diabetes. However, there were no students whose HbA1c level was more than 6.5%, and the incidence of childhood-onset type 1 diabetes in Japan is low (2.25/100,000 persons) (52) compared with most European countries and the US. Sixth, given the somewhat large interaction p-value, studies with a larger sample size would be needed to confirm our findings. Finally, this study is a cross-sectional study and does not clarify the causation between skipping breakfast and prediabetes in adolescents. In the future, longer duration randomized controlled trials and longitudinal studies from preschool children to adolescents are needed. In addition, it is necessary to evaluate the impact of skipping breakfast on prediabetes in other races to generalize our findings because there are racial differences in insulin sensitivity and insulin response (19). Analysis using indices of insulin sensitivity and insulin resistance calculated by fasting blood glucose and fasting insulin levels would also be helpful.

In summary, we found that skipping breakfast was associated with prediabetes after adjusting for the students’ demographic, lifestyle, and socioeconomic status, and this association was stronger among students with overweight. Our findings suggest that avoiding skipping breakfast may help to prevent prediabetes, especially for people with overweight.

## Data availability statement

The raw data supporting the conclusions of this article will be made available by the authors, without undue reservation.

## Ethics statement

The studies involving human participants were reviewed and approved by the Ethics Committee at the National Center for Child Health and Development (Study ID: 1147) and Tokyo Medical and Dental University (Study ID: M2016-284). Written informed consent to participate in this study was provided by the participants’ legal guardian/next of kin.

## Author contributions

TF conceived the study. TF, MO, AI and SD conducted the survey and collected data. KM was primarily responsible for data analysis and wrote the first draft of paper. NN and TF reviewed and edited the manuscript. All authors contributed to the article and approved the submitted version.
