# Supplementary material for: Association between skipping breakfast and prediabetes among adolescence in Japan: Results from A-CHILD study
Source: Front Endocrinol (Lausanne). 2023 Feb 22;14:1051592. doi: 10.3389/fendo.2023.1051592 (PMC9992887; doi:10.3389/fendo.2023.1051592)
Supplement: Supplementary file 1 [file DataSheet_1.docx]

Supplementary table 1. Odds ratio for prediabetes and skipping breakfast (n=1510)

|  |  | Adjusted model | |  |
| --- | --- | --- | --- | --- |
|  |  | OR | 95%CI | VIF |
| Frequency of breakfast | Everyday | Ref |  |  |
|  | Sometimes/rarely/never | **2.01** | **(1.04, 3.89)** | 1.04 |
| Child sex | Boy | Ref |  |  |
|  | Girl | 0.56 | (0.31, 1.01) | 1.06 |
| Annual household income (million yen) | < 3 | Ref |  |  |
|  | 3-6 | 1.41 | (0.45, 4.37) | 2.45 |
|  | 6-10 | 3.07 | (1.06, 8.93) | 2.51 |
|  | ≥ 10 | 1.39 | (0.34, 5.72) | 1.61 |
|  | Unknown/Missing | 0.89 | (0.22, 3.65) | 1.87 |
| Family history of diabetes | No | Ref |  |  |
|  | Yes | 1.29 | (0.44, 3.80) | 1.01 |
| BMI | <-1SD | 0.81 | (0.38, 1.72) | 1.06 |
|  | -1SD to 1SD | Ref |  |  |
|  | ≥1SD | 0.99 | (0.47, 2.09) | 1.08 |
| Wake-up time on weekdays | - 7:00 a.m. | Ref |  |  |
|  | 7:00 - 8:00 a.m. | 1.10 | (0.61, 1.98) | 1.18 |
|  | 8:00 a.m. - | 1.60 | (0.17, 14.69) | 1.05 |
| Wake-up time on weekends | - 7:00 a.m. | Ref |  |  |
|  | 7:00 - 8:00 a.m. | 0.60 | (0.27, 1.35) | 1.70 |
|  | 8:00 a.m. - | 0.57 | (0.28, 1.16) | 1.97 |
| Frequency of exercise | <1times/week | Ref |  |  |
|  | 1-2 times/week | 0.77 | (0.36, 1.66) | 1.36 |
|  | ≥3times/week | 0.71 | (0.37, 1.35) | 1.45 |
| Year | 2016 | Ref |  |  |
|  | 2018 | 0.94 | (0.48, 1.83) | 1.2 |
|  | 2020 | 0.83 | (0.42, 1.66) | 1.21 |

OR Odds ratio; CI confidence interval; VIF Variance Inflation Factor

Adjusted model: Adjusted for child sex, annual household income, family history of diabetes, BMI, wake-up time on weekdays and weekends, frequency of exercise and survey year

Supplementary table 2. Characteristics of participants stratified by year

| Year |  | 2016 | | | 2018 | | | 2020 | | |
| --- | --- | --- | --- | --- | --- | --- | --- | --- | --- | --- |
|  |  | Total | Frequency of breakfast | | Total | Frequency of breakfast | | Total | Frequency of breakfast | |
|  |  |  | Every day | Sometimes/ rarely/never |  | Every day | Sometimes/ rarely/never |  | Every day | Sometimes/ rarely/never |
|  |  | (N=483) | (N=405; 83.9%) | (N=78; 16.1%) | (N=525) | (N=435; 82.9% | (N=90; 17.1%) | (N=502) | (N=422; 84.1%) | (N=80; 15.9%) |
|  |  | N (%) | N (%) | N (%) | N (%) | N (%) | N (%) | N (%) | N (%) | N (%) |
| Prediabetes | HbA1c<5.6 | 464 (96.1%) | 394 (97.3%) | 70 (89.7%) | 504 (96.0%) | 415 (95.4%) | 89 (98.9%) | 484 (96.4%) | 409 (96.9%) | 75 (93.8%) |
|  | HbA1c≧5.6 | 19 (3.9%) | 11 (2.7%) | 8 (10.3%) | 21 (4.0%) | 20 (4.6%) | 1 (1.1%) | 18 (3.6%) | 13 (3.1%) | 5 (6.2%) |
| Child Sex | Boy | 237 (49.1%) | 211 (52.1%) | 26 (33.3%) | 253 (48.2%) | 214 (49.2%) | 39 (43.3%) | 267 (53.2%) | 226 (53.6%) | 41 (51.2%) |
|  | Girl | 246 (50.9%) | 194 (47.9%) | 52 (66.7%) | 272 (51.8%) | 221 (50.8%) | 51 (56.7%) | 235 (46.8%) | 196 (46.4%) | 39 (48.4%) |
|  | Missing | 0 (0%) | 0 (0%) | 0 (0%) | 0 (0%) | 0 (0%) | 0 (0%) | 0 (0%) | 0 (0%) | 0 (0%) |
| Annual household income  (million yen) | < 3 | 63 (13.0%) | 47 (11.6%) | 16 (20.5%) | 55 (10.5%) | 42 (9.7%) | 13 (14.4%) | 67 (13.3%) | 52 (12.3%) | 15 (18.8%) |
|  | 3 - 6 | 171 (35.4%) | 141 (34.8%) | 30 (38.5%) | 158 (30.1%) | 137 (31.5%) | 21 (23.3%) | 142 (28.3%) | 122 (28.9%) | 20 (25.0%) |
|  | 6 - 10 | 160 (33.1%) | 143 (35.3%) | 17 (21.8%) | 169 (32.2%) | 147 (33.8%) | 22 (24.4%) | 171 (34.1%) | 144 (34.1%) | 27 (33.8%) |
|  | ≥ 10 | 38 (7.9%) | 36 (8.9%) | 2 (2.6%) | 46 (8.8%) | 40 (9.2%) | 6 (6.7%) | 53 (10.6%) | 46 (10.9%) | 7 (8.8%) |
|  | Unknown/Missing | 51 (10.6%) | 38 (9.4%) | 13 (16.7%) | 97 (18.5%) | 69 (15.9%) | 28 (31.1%) | 69 (13.7%) | 58 (13.7%) | 11 (13.8%) |
| Family history of diabetes | No | 456 (94.4%) | 381 (94.1%) | 75 (96.2%) | 509 (97.0%) | 422 (97.0%) | 87 (96.7%) | 475 (94.6%) | 401 (95.0%) | 74 (92.5%) |
|  | Yes | 27 (5.6%) | 24 (5.9%) | 3 (3.8%) | 16 (3.0%) | 13 (3.0%) | 3 (3.3%) | 27 (5.4%) | 21 (5.0%) | 6 (7.5%) |
|  | Missing | 0 (0%) | 0 (0%) | 0 (0%) | 0 (0%) | 0 (0%) | 0 (0%) | 0 (0%) | 0 (0%) | 0 (0%) |
| BMI | <-1SD | 83 (17.2%) | 74 (18.3%) | 9 (11.5%) | 99 (18.9%) | 86 (19.8%) | 13 (14.4%) | 80 (15.9%) | 67 (15.9%) | 13 (16.2%) |
|  | -1SD to 1SD | 304 (62.9%) | 260 (64.2%) | 44 (56.4%) | 361 (68.8%) | 297 (68.3%) | 64 (71.1%) | 326 (64.9%) | 276 (65.4%) | 50 (62.5%) |
|  | ≥1SD | 83 (17.2%) | 61 (15.1%) | 22 (28.2%) | 56 (10.7%) | 44 (10.1%) | 12 (13.3%) | 89 (17.7%) | 73 (17.3%) | 16 (20.0%) |
|  | Missing | 13 (2.7%) | 10 (2.5%) | 3 (3.8%) | 9 (1.7%) | 8 (1.8%) | 1 (1.1%) | 7 (1.4%) | 6 (1.4%) | 1 (1.2%) |

Supplementary table 3. Characteristics of participants (N=1510)

|  |  | Total | Frequency of breakfast | |
| --- | --- | --- | --- | --- |
|  |  |  | Every day | Sometimes/rarely/never |
|  |  | (n=1510) | (n=1262; 83.6%) | (n=248; 16.4%) |
|  |  | n (%) | n (%) | n (%) |
| Wake-up time on weekdays | - 7:00 a.m. | 777 (51.5%) | 687 (54.4%) | 90 (36.3%) |
|  | 7:00 - 8:00 a.m. | 711 (47.1%) | 564 (44.7%) | 147 (59.3%) |
|  | 8:00 a.m. - | 13 (0.9%) | 4 (0.3%) | 9 (3.6%) |
|  | missing | 9 (0.6%) | 7 (0.6%) | 2 (0.8%) |
| Wake-up time on weekends | - 7:00 a.m. | 290 (19.2%) | 262 (20.8%) | 28 (11.3%) |
|  | 7:00 - 8:00 a.m. | 334 (22.1%) | 287 (22.7%) | 47 (19.0%) |
|  | 8:00 a.m. - | 873 (57.8%) | 701 (55.5%) | 172 (69.4%) |
|  | missing | 13 (0.9%) | 12 (1.0%) | 1 (0.4%) |
| Sleep duration on weekdays (hours) | ≤6 | 141 (9.3%) | 104 (8.2%) | 37 (14.9%) |
|  | 7 | 518 (34.3%) | 418 (33.1%) | 100 (40.3%) |
|  | 8-10 | 819 (54.2%) | 713 (56.5%) | 106 (42.7%) |
|  | ≥11 | 11 (0.7%) | 11 (0.9%) | 0 (0.0%) |
|  | missing | 21 (1.4%) | 16 (1.3%) | 5 (2.0%) |
| Sleep duration on weekends (hours) | ≤6 | 65 (4.3%) | 54 (4.3%) | 11 (4.4%) |
|  | 7 | 167 (11.1%) | 144 (11.4%) | 23 (9.3%) |
|  | 8-10 | 1168 (77.4%) | 972 (77.0%) | 196 (79.0%) |
|  | ≥11 | 88 (5.8%) | 73 (5.8%) | 15 (6.0%) |
|  | missing | 22 (1.5%) | 19 (1.5%) | 3 (1.2%) |
| Frequency of exercise | <1time/week | 477 (31.6%) | 376 (29.8%) | 101 (40.7%) |
|  | 1-2 times/week | 347 (23.0%) | 302 (23.9%) | 45 (18.1%) |
|  | ≥3times/week | 675 (44.7%) | 577 (45.7%) | 98 (39.5%) |
|  | missing | 11 (0.7%) | 7 (0.6%) | 4 (1.6%) |

Supplementary table 4. Odds ratio for skipping breakfast and wake-up time and sleep duration on weekdays and weekends and frequency of exercise (n=1510)

|  |  | OR | 95%CI |
| --- | --- | --- | --- |
| Wake-up time on weekdays | - 7:00 a.m. | Ref |  |
|  | 7:00 - 8:00 a.m. | **1.99** | **(1.50, 2.65)** |
|  | 8:00 a.m. - | **17.15** | **(5.18, 56.92)** |
| Wake-up time on weekends | - 7:00 a.m. | Ref |  |
|  | 7:00 - 8:00 a.m. | 1.53 | (0.93, 2.52) |
|  | 8:00 a.m. - | **2.30** | **(1.50, 3.51)** |
| Sleep duration on weekdays (hours) | ≤6 | Ref |  |
|  | 7 | 0.67 | (0.44, 1.04) |
|  | 8-10 | **0.42** | **(0.27, 0.64)** |
|  | ≥11 | NA |  |
| Sleep duration on weekends (hours) | ≤6 | Ref |  |
|  | 7 | 0.78 | (0.36, 1.72) |
|  | 8-10 | 0.99 | (0.51, 1.93) |
|  | ≥11 | 1.01 | (0.43, 2.37) |
| Frequency of exercise | <1times/week | **1.58** | **(1.16, 2.15)** |
|  | 1-2 times/week | 0.88 | (0.60, 1.28) |
|  | ≥3times/week | Ref |  |

OR Odds ratio; CI confidence interval

Supplementary table 5. Odds ratio for prediabetes and skipping breakfast (n=1510)

|  |  | Adjusted model | |
| --- | --- | --- | --- |
|  |  | OR | 95%CI |
| Frequency of breakfast | Everyday | Ref |  |
|  | Sometimes/rarely/never | **1.98** | **(1.04, 3.79)** |
| Child sex | Boy | Ref |  |
|  | Girl | **0.55** | **(0.31, 0.97)** |
| Annual household income (million yen) | < 3 | Ref |  |
|  | 3-6 | 1.48 | (0.47, 4.61) |
|  | 6-10 | **3.14** | **(1.08, 9.18)** |
|  | ≥ 10 | 1.39 | (0.34, 5.77) |
|  | Unknown/Missing | 1.12 | (0.29, 4.29) |
| Family history of diabetes | No | Ref |  |
|  | Yes | 1.29 | (0.44, 3.84) |
| BMI | <-1SD | 0.82 | (0.39, 1.74) |
|  | -1SD to 1SD | Ref |  |
|  | ≥1SD | 0.96 | (0.46, 2.01) |
| Sleep duration on weekdays (hours) | ≤6 | Ref |  |
|  | 7 | 0.85 | (0.33, 2.18) |
|  | 8-10 | 1.00 | (0.39, 2.56) |
|  | ≥11 | NA |  |
| Sleep duration on weekends (hours) | ≤6 | Ref |  |
|  | 7 | 0.70 | (0.21, 2.30) |
|  | 8-10 | 0.45 | (0.16, 1.28) |
|  | ≥11 | 0.37 | (0.06, 2.13) |
| Year | 2016 | Ref |  |
|  | 2018 | 0.94 | (0.48, 1.83) |
|  | 2020 | 0.83 | (0.42, 1.66) |

OR Odds ratio; CI confidence interval

Adjusted model: Adjusted for child sex, annual household income, family history of diabetes, BMI, sleep duration on weekdays and weekends and survey ye
